# Further observations on the relationship between the FGFR4 Gly388Arg polymorphism and lung cancer prognosis

**DOI:** 10.1038/sj.bjc.6603816

**Published:** 2007-05-22

**Authors:** A Matakidou, R el Galta, M F Rudd, E L Webb, H Bridle, T Eisen, R S Houlston, and the GELCAPS Consortium

**Affiliations:** 1 Section of Cancer Genetics, Institute of Cancer Research, Surrey, UK; 2 Department of Oncology, University of Cambridge, Cambridge, UK

**Keywords:** lung cancer, FGFR4, Gly388Arg polymorphism, prognosis

## Abstract

The Gly388Arg polymorphism in the fibroblast growth factor receptor 4 (*FGFR4*) gene has been reported to influence prognosis in a wide variety of cancer types. To determine whether Gly388Arg is a marker for lung cancer prognosis, we genotyped 619 lung cancer patients with incident disease and examined the relationship between genotype and overall survival. While we employed a comprehensive set of statistical tests, including those sensitive to the detection of differences in early survival, our data provide little evidence to support the tenet that the *FGFR4* Gly388Arg polymorphism is a clinically useful marker for lung cancer prognosis.

Lung cancer remains a leading cause of cancer death worldwide (Parkin *et al*, 2005). Despite recent improvements in treatment, the prognosis has only marginally improved and 5-year survival rates from both small-(SCLC) and non-small-cell lung cancer (NSCLC) are generally no better than 15% (Cancer Research UK). While the major prognostic determinant is stage at presentation, there is variability in survival for patients with same-stage disease, making it highly advantageous to identify further prognostic markers, which may predict patients likely to benefit from treatment. Furthermore, detecting genes with prognostic relevance has the potential to aid the identification of pathways that may be targeted for therapeutic interventions.

The FGF/FGFR receptor (FGFR) signalling pathway plays a pivotal role in cellular biology, being involved in differentiation, angiogenesis and motility (reviewed in Powers *et al* (2000)). Dysregulation of this pathway is a feature of a number of tumours and allelic imbalance at several FGF/FGFR loci is common in lung cancer. Correlations between such changes and lymph node status in cancer has been reported (Beau-Faller *et al*, 2003), implying that FGF/FGFR signalling may play an important role in the growth and survival of cancer cells.

A specific role for *FGFR4* in cancer is not well established, but altered expression has been documented in breast, lung, pancreatic and prostate cancers (Bange *et al*, 2002; Shah *et al*, 2002; Nakamura *et al*, 2004; Wang *et al*, 2004). Recently, a common polymorphism in the transmembrane domain of the FGFR4 gene, Gly388Arg, has been reported to correlate with tumour aggressiveness (lymph node metastasis, advanced stage at presentation and reduced survival in several cancer types, including breast, sarcoma, lung and prostate (Bange *et al*, 2002; Morimoto *et al*, 2003; Nakamura *et al*, 2004; Wang *et al*, 2004; Spinola *et al*, 2005a)). Some of these studies were, however, conducted on relatively small sample sizes and subsequent studies of breast, colon, head and neck, and bladder cancers (Becker *et al*, 2003; Jezequel *et al*, 2004; Streit *et al*, 2004; Spinola *et al*, 2005b; Yang *et al*, 2006), have provided little support for an association between *FGFR4* Gly388Arg genotype and prognosis.

To evaluate the prognostic significance of the *FGFR4* Gly388Arg polymorphism in lung cancer, we analysed a cohort of 619 lung cancer patients. We employed a comprehensive set of statistical tests, including those sensitive to detecting differences in early survival.

## MATERIALS AND METHODS

### Patients

Patients with lung cancer were ascertained through the Genetic Lung Cancer Predisposition Study (GELCAPS), a population-based study of lung cancer. Further details about the design and conduct of the study are described in previously published material (Matakidou *et al*, 2005). The current analysis is based on 619 female patients from whom detailed clinicopathological data and follow-up information on patients had been collected using a standardised proforma. All cases in this current analysis are white Caucasians who lived in the United Kingdom at the time of sample acquisition. The clinicopathologic characteristics of the patients are detailed in Table 1. DNA was extracted from EDTA-venous blood samples by a salt extraction procedure and quantified by Picogreen (Invitrogen, Paisley, UK). Genotyping was conducted by means of Illumina Sentrix Bead Arrays (Illumina, San Diego, CA, USA) according to the manufacturer's protocols (details available on request). Ethical approval for the study was obtained from the London Multi-Centre Research Ethics Committee (MREC/98/2/67) in accordance with the tenets of the Declaration of Helsinki. All participants provided informed consent.

### Statistical methods

Statistical analyses were undertaken using S-Plus (Version 8, Insightful Corporation, Seattle, WA, USA). We tested for Hardy–Weinberg equilibrium using an exact test based on genotypic frequencies. Overall survival (OS) of patients was the end point of the analysis. Survival time was calculated from the date of diagnosis of lung cancer to the date of death. Patients who were not deceased were censored at the date of last contact. Mean follow-up time was computed among censored observations only. Kaplan–Meier survival curves according to genotype were generated and the homogeneity of the survival curves between genotypes was evaluated using the log-rank, and Wilcoxon and Fleming–Harrington tests (with ρ set to 0, 1 and −1 within the S-Plus *servdiff* function for each of the tests respectively) (Klein and Moeschberger, 1997). The log-rank test is usually the preferred test, but the other tests were conducted to show the influence of the polymorphic variation at different times of follow-up in order to detect any difference in early and late stages of disease (Klein and Moeschberger, 1997). Cox regression analysis (Klein and Moeschberger, 1997) was used to estimate hazard ratios (HRs) and their 95% confidence intervals (CI), while adjusting for age, smoking, treatment, histology and stage.

## RESULTS

One hundred and fifty-four of the total number of patients (25%) had SCLC, less than half (43%) presenting with limited disease. Of the 465 patients with NSCLC, 57 (13%) had stage I, 68 (15%) had stage II, 196 (43%) had stage III and 130 (29%) had stage IV disease at diagnosis. The majority of patients with limited-stage SCLC had been treated with a combination of radical radiotherapy and chemotherapy, while all patients received chemotherapy (Table 1). The main treatment modality for SCLC patients with extensive disease was chemotherapy. Patients with early-stage NSCLC (stage I and II disease) were mainly treated with surgical resection of the primary tumour, while about one-third received chemotherapy and radical radiotherapy. The mainstay treatment modality of patients with stage III and IV NSCLC was chemotherapy. The mean follow-up time for patients alive at end of follow-up was 21.0 months (range 0–60.5 months). There were 389 (62.8%) deaths in the entire cohort. For all patients, the median survival time (MST) was 16.2 months. Patients with SCLC had an MST of 17.8 and 11.1 months, if diagnosed with limited and extensive disease, respectively. For NSCLC, by stage, MST ranged from 11.5 months in stage IV patients to 49.2 months in the stage I group. As these survival rates are not significantly different to those documented in previously published audits of lung cancer prognosis, there is no evidence that ‘healthy study participant’ selection will have biased our analyses.

Surgery, any chemotherapy and treatment specifically with platinum-based compounds did not satisfy the proportional hazards assumption required for the Cox model. Therefore, we used a stratified Cox proportional hazards model, stratifying on surgery, platinum, chemotherapy and surgery. As expected, factors significantly influencing patient prognosis were stage at presentation (*P*<10^−4^), histology (*P*=0.026) and radiotherapy (*P*=0.0042). Smoking, family history of lung cancer and age at diagnosis did not impact on survival.

**Figure 1:**
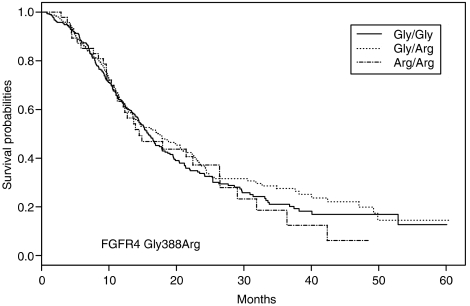
Kaplan–Meier curves for all lung cancer patients. Survival curves for the Gly/Gly homozygotes are shown as a solid line. The dashed line depicts the survival curve for the Gly/Arg heterozygotes, and the broken line depicts the survival curve for the rare homozygotes Arg/Arg.

Genotyping of the Gly388Arg polymorphism in all patients showed 319 (51.5%) were homozygous Gly/Gly alleles, 252 were heterozygous for the Gly/Arg alleles (40.7%) and 48 (7.8%) homozygous for the Arg/Arg alleles. There was no evidence in the data set indicative of Hardy–Weinberg disequilibrium (*P*=0.92). There was no correlation between *FGFR4* SNP genotype and the pathological parameters, stage (*P*=0.58) and histology (*P*=0.94). Figure 1 shows Kaplan–Meier survival analysis by genotype. All non-parametric tests provided no evidence for a relationship between SNP genotype and OS (*P*-values 0.555, 0.751 and 0.347 for log rank, Wilcoxon and Fleming–Harrington test statistics, respectively). Under the Cox proportional hazards model, the HRs for Arg/Gly heterozogosity, Arg/Arg homozygosity and Arg-carrier status were: 0.95 (95% CI: 0.77–1.18), 1.06 (95% CI: 0.72–1.56) and 0.97 (95% CI: 0.79–1.19), respectively. A stratified analysis based on individual histological groupings also provided no evidence that SNP genotype influenced OS (Table 2).

## DISCUSSION

We have found no evidence of an association between *FGFR4* Gly388Arg genotype and lung cancer pathological parameters. Furthermore, our findings do not indicate that the *FGFR4* genotype is an independent predictor of prognosis for lung cancer. Stratification by histological subtype did not impact on the significance of these results. Our observations are in contrast with those reported by Spinola *et al* (2005a), supporting a correlation between *FGFR4* genotype and lung cancer prognostic variables in patients with adenocarcinomas.

The major strengths of our study are its large size, the fact that it is population based on patients with incident disease and that it has involved the systematic follow-up of patients. Even though bias from non-uniform treatment is a potential confounder in studies of some solid tumours, the management of lung cancer is relatively uniform in the United Kingdom, as there are only a restricted number of effective chemotherapeutic agents and prognosis is uniformly poor. Support for this assertion is provided by the fact that the survival rates observed in our patient cohort were not different to those expected. It is therefore unlikely that any spurious influence as a consequence of study design will have impacted significantly on findings. As our analysis was restricted to white patients and there was no statistical evidence for population substructure, findings are unlikely to be confounded by this form of potential bias.

Our study does not support the findings of Spinola *et al* (2005a). This could in part be a consequence of differences in study design. Our study included a higher proportion of patients with late-stage disease, with consequent shorter follow-up time and potential reduced power to demonstrate a relationship. Furthermore, our analysis was not restricted to adenocarcinoma cases. Accepting this caveat, we did not find evidence of a relationship between prognosis and *FGFR4* genotype for any of the lung cancer histology.

The notion that germline variation in *FGFR4* may influence biology of tumours is an attractive postulate. The reason for the conflicting results between studies that have evaluated the role of Gly388Arg is not entirely obvious. Some differences may reflect tissue-specific effects of this polymorphism. It is, however, not uncommon for the first published studies to report over-inflated estimates of effects, which subsequent larger studies cannot replicate. Studies that have examined the role of FGFR4 in the carcinogenesis provide evidence for the complexity of the FGF/FGFR signalling pathway in different tumour types (Olson *et al*, 1998; Cavallaro *et al*, 2001; Ezzat *et al*, 2002; Shah *et al*, 2002). It therefore seems unlikely *a priori* that a single SNP, albeit one with functional effects, will impart substantial differences in cancer prognosis independently. In conclusion, we believe there is currently little evidence to suggest that the Gly388Arg polymorphism of FGFR4 represents a robust marker of lung cancer prognosis.

## Figures and Tables

**Table 1:** Demographic and follow-up characteristics of patients

	**Number of patients (%)**
Total	619
	
Mean age (years)	64.8
	
*Smoking habits*	
Non-smokers	49 (8)
Smokers	570 (92)
	
*Histology*	
Small cell (SCLC)	154 (25)
Non-small cell (NSCLC)	465 (75)
Squamous	180 (30)
Adenocarcinoma	164 (27)
	
*Tumour stage (by histology)*	
*SCLC*	
Limited	66 (43)
Extensive	86 (57)
	
*NSCLC*	
I	57 (13)
II	68 (15)
III	196 (43)
IV	130 (29)
	
Median survival time (months)	16.2
Events (deaths)	389 (62.8)
	
*Median survival time, months, by histology and stage*	
*SCLC*	
Limited	17.8
Extensive	11.1
All stages	13.5
	
*NSCLC*	
I	49.2
II	31.9
III	16.2
IV	11.5
All stages	17.6

NSCLC=non-small-cell lung cancer; SCLC=small-cell lung cancer.

**Table 2:** Relationship between OS from lung cancer and *FGFR4* Gly388Arg genotype

	**Genotype**	**Count**	**HR (95% CI)**	***P*-value**
*All cancer*				
	Gly/Gly	319		
	Gly/Arg	252	0.95 (0.77–1.18)	0.67
	Arg/Arg	48	1.06 (0.72–1.56)	0.76
	Gly/Arg and Arg/Arg		0.97 (0.79–1.19)	0.78
	Log rank=1.2, d.f.=2; *P*=0.56			
NSCLC				
	Gly/Gly	238		
	Gly/Arg	191	0.88 (0.68–1.13)	0.31
	Arg/Arg	36	1.17 (0.75–1.82)	0.50
	Gly/Arg and Arg/Arg		0.92 (0.72–1.17)	0.48
	Log rank=1.7, d.f.=2; *P*=0.43			
				
SCLC				
	Gly/Gly	81		
	Gly/Arg	61	1.18 (0.77–1.80)	0.44
	Arg/Arg	12	0.88 (0.39–1.95)	0.74
	Gly/Arg and Arg/Arg		1.12 (0.75–1.69)	0.58
	Log rank=2, d.f.=2; *P*=0.37			
				
Adenocarcinoma				
	Gly/Gly	83		
	Gly/Arg	71	0.93 (0.60–1.45)	0.75
	Arg/Arg	10	1.07 (0.50–2.34)	0.85
	Gly/Arg and Arg/Arg		0.95 (0.62–1.45)	0.83
	Log rank=3.8, d.f.=2; *P*=0.15			

CI=confidence interval; HR=hazard ratio; NSCLC=non-small-cell lung cancer; OS=overall survival; SCLC=small-cell lung cancer.
